# Coronavirus Disease (COVID-19) in the Middle East: A Call for a Unified Response

**DOI:** 10.3389/fpubh.2020.00209

**Published:** 2020-05-19

**Authors:** Tania Sawaya, Tala Ballouz, Hassan Zaraket, Nesrine Rizk

**Affiliations:** ^1^Division of Biology and Medicine, Undergraduate Program in Biology, Department of Molecular Microbiology and Immunology, Brown University, Providence, RI, United States; ^2^Department of Public Health, Epidemiology Biostatistics and Prevention Institute, University of Zurich, Zurich, Switzerland; ^3^Department of Experimental Pathology, Immunology, and Microbiology, Center of Infectious Disease, Faculty of Medicine, American University of Beirut, Beirut, Lebanon; ^4^Division of Infectious Diseases, Department of Internal Medicine, Faculty of Medicine, American University of Beirut Medical Center, Beirut, Lebanon

**Keywords:** COVID-19, SARS-CoV-2, Middle East, Arab league, pandemic preparedness and response, coronavirus disease

The Middle East—traditionally considered to be composed of the Arab-speaking countries in the Eastern Mediterranean region in addition to Iran—has continuously been considered a hotspot for infectious diseases (1). In addition to cross-border travel, its countries attract millions of international travelers each year for tourism, business, and pilgrimage. Furthermore, it remains an area of political turmoil and economic instability. Middle Eastern nations vary greatly in resources, growth indices, and economic strengths (2). For that, budget gaps (3) and difficulty in securing essential resources could compromise the response to an infectious disease outbreak. Violent conflicts have weakened the health infrastructure of several countries in the region and displaced millions of people. The densely populated refugee camps are particularly worrying. Poor hygiene measures and fragmented access to healthcare (4) have rendered these populations more vulnerable to disease and left out of pandemic preparedness (5, 6). It is also worth noting that Middle Eastern populations have high rates of diabetes (7) and cardiovascular problems (8) that have been found to be risk factors for severe COVID-19 disease (9). For all of those reasons, the spread of COVID-19 in the region is particularly alarming and invites serious dialogue, transparency, and cooperation.

In March 2020, the WHO declared the novel coronavirus outbreak a pandemic. It has since triggered a global lockdown and disrupted international travel, causing an unprecedented economic crisis (10). The Middle East witnessed a rapid rise in the number of confirmed cases with Iran emerging as its epicenter. This outbreak comes as yet another striking reminder that the preparedness of Middle Eastern countries' healthcare systems for emerging global infectious diseases is still lagging (11). In addition, there have been no clear strategies or plans shared by countries in the region to suggest a coordinated response. The Arab League as an entity has not addressed this issue and pre-scheduled meetings were postponed due to the evolving COVID-19 situation. This appears as a missed opportunity for collaboration and cooperation between countries that share a lot more than borders.

Although there has not been a sign of a coordinated region-wide response yet, affected countries in the Middle East are trying to contain the outbreak individually. The response of those countries was variable and largely dictated by the mounting fear and rapid expansion of the COVID-19 outbreak worldwide (12). The rapid surge of COVID-19 in Iran was overwhelming and contributed to fueling the epidemic in nine neighboring countries and Canada (13, 14). Although initially criticized for underreporting cases, Iran's cooperation with the WHO's Regional Office for the Eastern Mediterranean (EMRO) seems to have been productive in making the reports more timely (15). The country's capacity to deal with this pandemic, though, like many others' in the region, is limited (16). More recently, Egypt faced a rapid surge in the number of infections and deaths as public health measures were late to be introduced (17, 18). At the time of writing this piece, several countries are still reporting an increase in the number of cases, probably reflecting increased testing capacity (3, 19). In fact, many of the Gulf Cooperation Council's (GCC) countries have taken notable measures to increase testing and rank among the highest countries in the world in terms of tests per million inhabitants (20). However, concerns are high regarding a potential outbreak among migrant workers, most of whom live in substandard, and crowded conditions (12).

The WHO-EMRO developed a regional strategic preparedness and response plan to aid Middle Eastern countries in building capacity to respond to the COVID-19 pandemic at an attempt to build a coordinated response (3). Decisions to close borders and restrict travel from affected nations were taken by many countries in the region, albeit considered delayed by some (21). At this point, most countries are under lockdown and are only admitting flights in the case of repatriation, cargo, or humanitarian aid. Some nations have also banned the exportation of medical supplies to ensure local demand is met (3). Countries such as Lebanon and the UAE took early measures to contain the outbreak, enforcing school closures, and other forms of social distancing. Authorities in Saudi Arabia canceled Umrah pilgrimage and access to Mecca to non-residents in an effort to contain the rapidly-spreading virus (22). Now with the start of Ramadan, the regional, and local agencies are also promoting safe practices (3). Governments and the WHO have resorted to TV and social media outlets and even mobile operators (23) to spread awareness and promote physical distancing while fighting the “infodemic” throughout the Middle East.

It is worth noting that the region had already witnessed outbreaks of the two other novel coronaviruses, SARS-CoV and MERS-CoV, as well as the 2009 H1N1 pandemic. Affected countries had consequently learned some lessons in terms of handling outbreaks (24, 25). For example, shortly after the discovery of MERS-CoV, the Saudi ministry of health established the Command and Control System and the Saudi Center for Disease Control and Prevention. Today, they lead the country's frontline response to SARS-CoV-2. Other countries like Qatar put together emergency preparedness plans that have effectively reduced the number of MERS-CoV cases (26) over the last 8 years. Most of the countries in the region also established national influenza centers following the 2009 H1N1 pandemic, many of which were supported by the WHO's Pandemic Influenza Preparedness Framework (PIPF). More recently, Dubai has emerged as the transportation hub for WHO relief efforts, handling over 88% of worldwide aide shipments. Kuwait also topped the WHO COVID-19 relief fund donors list with over 40 million USD (3). Thus, the Middle East possesses different levels of expertise, frontline experience, logistical skills, and financial resources that, if shared, could be greatly beneficial for its populations and ramp up the effectiveness of its countries' prevention and response plans. So why has that cooperation not happened yet? And how different would the situation be if countries had worked together from the start?

The political divides are probably the main obstacle to more collaboration among Middle Eastern countries as tensions and polarization weigh on diplomatic relationships. The Gulf Cooperation Council**—**comprising the UAE, KSA, Oman, Kuwait, Qatar, and Bahrain**—**has emerged as best prepared to respond to this pandemic as argued for above, while countries like Yemen (27), Libya and Syria, and territories like the Gaza strip (28, 29) are nowhere near that capacity. Local protests were also undergoing in Iraq and Lebanon when confinement orders to curb the spread of the virus were issued. The variation in the number of cases between countries may also reflect the discrepancies in preparedness and response. For example, while Saudi Arabia has had more than 25,000 cases so far, Syria has reported only 44 cases. Similarly, the case fatality rate (CFR) is highly variable around the region, ranging from 0.2% in Djibouti to 7.0% in Sudan and Syria (excluding Yemen because of statistical unreliability) (19). It is also worth noting that, of all confirmed cases reported to the WHO-EMRO, only 3.4% have been documented in detail (3). This could seriously impede research in the region and discourage cooperation.

The region as a whole should help secure a budget to fund prevention and response efforts for internally displaced people, refugees, and migrant workers. In addition, a collective investment in telehealth or other methods to ensure care for chronic conditions for the length pandemic could be the base for a fruitful collaboration (30). Finally, detailed COVID-19 case report submissions to the WHO should drastically increase. This would secure a solid database for regional and local experts' research endeavors that will consequently inform policy and accurately document the epidemiology of COVID-19 in the Middle East. As most countries are studying strategies to re-open businesses and borders, regional cooperation may provide the necessary exchange of expertise, and resources to halt the virus' spread, and allow for a safe resumption of human activity. With no vaccine or approved treatment on the horizon, the COVID-19 pandemic is expected to linger, forcing countries everywhere to adapt to the new normal.

Although it may be too late to build a unified response for all of the Middle East it might not be too late to show some coordination**—**especially for richer states to provide logistical, technical, and financial assistance to their neighbors. Public health initiatives might be more effective if coordinated within subregions of the MENA namely the Maghreb (North Africa), Mashreq (Levant), and Gulf since they have more similar healthcare systems and demographics (31). The role of the WHO EMRO has been instrumental in bridging gaps and regional readiness. Aggressive containment efforts and significant public engagement should be urgently mobilized in the hopes of disrupting the spread of COVID-19 in the Middle East. No country is isolated from the other, so cooperation and coordination would only be beneficial in preventing a possible public health catastrophe. If we won't unite now, then when?

## Author Contributions

NR conceived the presented idea. NR and TS wrote the manuscript with contribution from TB and HZ. All authors commented on and contributed to the shaping of the manuscript.

## Conflict of Interest

The authors declare that the research was conducted in the absence of any commercial or financial relationships that could be construed as a potential conflict of interest.
